# The effects of age-related macular degeneration on quality of life in a Brazilian population

**DOI:** 10.1186/s40942-021-00290-z

**Published:** 2021-03-16

**Authors:** Alicia Buffoni Roque, Géssica Fernandes da Silva Borges, Ricardo Yuji Abe, Osias Francisco de Souza, Maria Cecília Machado, Tamara Ferreira, Newton Kara José, José Paulo Cabral de Vasconcellos

**Affiliations:** 1 Department of Ophthalmology, Faculty of Medical Science, University of Campinas, Campinas, SP Brazil; 2 Conderg-Divinolândia, Divinolândia, SP Brazil

**Keywords:** Quality of life, Age-related macular degeneration, Questionnaires

## Abstract

**Background:**

To evaluate the impact of age-related macular degeneration (AMD) on the quality of life (QoL) in a Brazilian population using The National Eye Institute-Visual Function Questionnaire-25 (NEI-VFQ-25).

**Methods:**

This observational study included 462 participants from the Departments of Ophthalmology of the University of Campinas and Conderg-Divinolândia. The NEI-VFQ-25 questionnaire and Rasch analysis were used to assess the vision-related quality of life (VRQoL). Patients with macular neovascularization were interviewed at enrollment and after three loading doses of intravitreal anti-vascular endothelial growth factor (anti-VEGF) treatment.

**Results:**

One hundred thirty-three patients were excluded because they had another ophthalmic disease, for a total of 349 patients included in the study (177 in the AMD group, 172 in the control group; 56.4% were women; mean ± standard deviation age, 70.6 ± 9.5 years). Most NEI-VFQ-25 subscale scores were significantly lower in the AMD group compared with the control group. The Rasch-calibrated NEI-VFQ-25 median score in the visual-functioning component was 56.41 for the AMD group and 61.53 for the control group, a difference of ± 4.00 (P = 0.0001). Separate analyses of the sociodemographic and ocular characteristics showed that the NEI-VFQ-25 scores were affected mostly by family income, educational level, descent, diet (vegetables/fruits), physical activity, and visual acuity (VA). The longitudinal component assessed a different group of 48 patients with exudative disease treated with anti-VEGF drugs. The mean logarithm of the minimum angle of resolution change in VA in treated eyes was a 0.16 decrease (P = 0.01). The mean change in the optical coherence tomography macular thickness was a 36.74-μm decrease (P = 0.012) from baseline to 4 months. The mean NEI-VFQ-25 scores improved significantly from baseline to follow-up at 4 months in almost all subscales.

**Conclusions:**

In a Brazilian community, patients with AMD had a worse VRQoL than controls. The AMD severity and bilaterality were associated with decreased NEI-VFQ-25 scores. Higher family income, educational level, descent, and lifestyle significantly improved several subscales of the NEI-VFQ-25 questionnaire. Treated patients with exudative AMD had improvements in the VA, macular thickness, and most NEI-VFQ-25 subscale scores.

## Background

Age-related macular degeneration (AMD) is a chronic progressive disease that mainly affects the central retina. It is estimated that 200 million people worldwide have AMD as of 2020, and this number is expected to reach 288 million in 2040 [1]. These numbers make AMD one of the leading causes of blindness in adults over 50 years of age, according to the World Health Organization [1]. Two forms of AMD, dry and wet, are clinically distinguished. In 1967, Gass provided the first description of the many pathophysiological features of neovascular AMD [2]. Over five decades, many studies have been performed based on imaging data from various imaging methods and have provided invaluable information [3, 4]. In 2019, Spaide et al. purposed a consensus classification of neovascular AMD based mainly on optical coherence tomography (OCT) and OCT angiography findings and classified wet AMD into macular neovascularization types I, II, and III, and dry AMD into the outer retinal atrophy and the retinal pigment epithelial atrophy.

Central vision, which directly affects a person’s ability to interact with the environment, is essential for performing daily routines. Central vision loss frequently leads to disability, dependency, impairment, and consequent emotional, functional, and psychological damage, while also reducing quality of life (QoL) [5, 6]. Patients with visual impairment caused by AMD frequently report depression [5, 7] and develop psychological issues similar to those experienced by patients with other serious chronic diseases, such as acquired immunodeficiency syndrome and chronic obstructive pulmonary disease [6].

The aspects of daily living activities and well-being are not usually assessed during a routine medical appointment. Therefore, patient-reported outcome measures obtained through questionnaires have been used to measure the effect of eye disorders on health-related QoL (HRQoL) and, more specifically, vision-related QoL (VRQoL) [8]. The National Eye Institute-Visual Function Questionnaire-25 (NEI-VFQ-25) was developed to measure QoL in people with eye diseases such as strabismus, cataract, glaucoma, and AMD [9–11]. This questionnaire has been translated into Portuguese and validated within Brazilian sociocultural contexts [12].

Several studies have investigated the effects of AMD on HRQoL, but few studies have been conducted in Latino populations [8, 13, 14]. This study measured the impact of AMD on VRQoL in Brazilian patients with AMD using the results of the NEI-VFQ-25 questionnaire calibrated with a Rasch analysis.

The primary endpoint of the current study was the impact of AMD on VRQoL in Brazilian patients and the effects of three initial doses of intravitreal anti-vascular endothelial growth factor (VEGF) treatment on VRQoL. The secondary endpoint was the association between sociodemographic variables and VRQoL.

## Methods

### Design and sample

This observational longitudinal study included 462 participants from the Departments of Ophthalmology of the University of Campinas and Conderg-Divinolândia from November 2017 to July 2018. This study was conducted to determine the effect of AMD on VRQoL. Three hundred and forty-nine patients were included in the analysis, of which 177 had AMD (AMD group) and 172 did not (control group).

In accordance with the Declaration of Helsinki, all participants provided written informed consent before taking part in the study. Patients were interviewed to obtain demographic data, which included history of ocular and medical conditions, self-reported race, educational level, income, ethnic backgrounds of both parents, marital status, sun exposure, smoking habits, systemic diseases, employment status, physical activities, alcohol consumption, and dietary habits.

AMD severity was defined using the Clinical Age-Related Maculopathy Staging (CARMS) [15]. This classification assigns scores from 1 to 5 as follows: 1, no drusen; 2, small drusen or foveal pigment irregularities; 3, intermediate disease; 4, geographic atrophy; and 5, exudative maculopathy. The AMD severity of each eye was rated independently from that of the contralateral eye. Patients with AMD grade 4 or 5 were considered to have severe disease. Because previous analyses have shown that QoL is more correlated with visual acuity (VA) in the eye with better version [16, 17], the patients were divided into three subgroups to assess their QoL: subgroup 1 included patients with at least one eye at stage 2 or 3, subgroup 2 included those with one eye at stage 2 or 3 and the other eye at stage 4 or 5, and subgroup 3 included those with both eyes at stage 4 or 5.

Two examiners determined the degree of AMD based on the fundus examination. The inclusion criteria for patients in the control group were the absence of AMD, both eyes being in stage 1 of the CARMS classification, and age greater than 50 years. For the AMD group, the inclusion criteria were age greater than 50 years and disease at stages 2 to 5 according to the CARMS classification. The exclusion criteria were the presence of glaucoma, cataracts of grade 2 or more according to the Lens Opacities Classification System (LOCS) III [18], and the presence of corneal opacities or any other retinal diseases.

Baseline testing included measurement of the best-corrected VA (BCVA), slit-lamp biomicroscopy, Goldmann applanation tonometry, dilated fundus evaluation using a double aspheric lens of + 78 diopters, fundus color photography, and swept-source OCT (Topcon DRI OCT Triton Plus, Topcon Medical Systems, Inc., Oakland, NJ, USA).

The NEI-VFQ-25 questionnaire was used to assess the VRQoL twice, first at enrollment and then after the third loading dose of intravitreal anti-VEGF treatment for those who needed treatment.

### Assessment of HRQoL

The NEI-VFQ-25 questionnaire was designed to cover domains related to vision and health that are relevant to patients with chronic eye disorders [19, 20]. This questionnaire comprises 25 questions divided into 12 subscales, which include one subscale related to general health and 11 vision-related subscales (general vision, ocular pain, near activities, distance activities, social functioning, mental health, role difficulties, dependency, driving, color vision, and peripheral vision). A composite score was reached based on the average of the 11 vision-related subscales.

Each subscale consists of a minimum of one and a maximum of four items. The algorithm used scores ranging from 0 to 100, with higher values representing better visual function in relation to the individual’s well-being. In this format, the scores represent a percentage of the possible total; in other words, a score of 50 equals 50% of the highest possible value.

### Statistical analyses

Exploratory data analyses were performed using descriptive statistics (mean, standard deviation, minimum, median, maximum, frequency, and percentage). Comparisons between the groups were performed using the Mann–Whitney, Kruskal–Wallis, chi-square, or Fisher’s exact tests. To evaluate the effect of clinical and demographic factors on patient QoL among the different stages of AMD, analysis of variance (ANOVA) was used and adjusted for the degree of AMD. The adopted significance level was 5%.

Comparisons between the pretreatment and posttreatment intravitreal results based on the VA, OCT macular thickness, and NEI-VFQ-25 questionnaire were assessed using the Wilcoxon test.

We also used the Rasch-calibrated NEI-VFQ-25 scores from the visual-functioning component separately from the socioemotional component [21]. Rasch analyses were performed using Winsteps 3.81.0 (Chicago, IL, USA) to obtain the final estimates of “person measures.” All statistical analyses were performed using the Statistical Analysis System for Windows, version 9.4 (SAS Institute Inc., Cary, NC, USA).

## Results

**Fig. 1:**
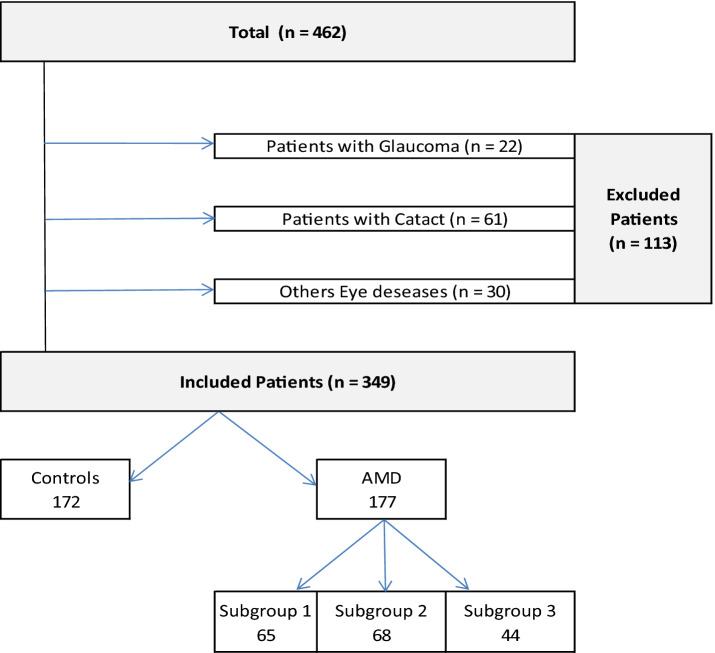
Flow chart of patient selection

Among the 462 patients studied, 133 were excluded because of glaucoma, cataracts, or other associated ophthalmic disease that could confound the results. We included 349 patients in the analysis, 177 with AMD (the AMD group) and 172 without AMD (the control group). In the AMD group, 68 patients were in subgroup 1, 65 in subgroup 2, and 44 in subgroup 3 (Fig. 1).

Of the 349 patients included in the analyses, 56.4% were women (mean ± standard deviation age, 70.6 ± 9.5 years). The mean age of the control patients was 68.8 ± 8.5 years, and the mean age of the patients with AMD was 72.3 ± 10.1 years. Analyses of the ages among the three AMD subgroups indicated that the average age was the highest in subgroup 3 (74.8 ± 11.0 years), followed by subgroup 2 (69.8 ± 9.4), and subgroup 1 (73.1 ± 9.8 years). This difference reached significance (p = 0.0067) (Table 1).

**Table 1:** Sociodemographic characteristics of the sample

Variable	Control	AMD	Total	p-value
Age				
Mean	68.8 (8.5)	72.3 (10.1)	70.6 (9.5)	0. 0007^a^
Median	69	73	70	
BEVA				
Mean	0.408 (0.252)	0.406 (0.437)	0.407 (0.358)	0.0040^a^
Median	0.400	0.300	0.300	
Gender				
Female	104 (60.5%)	93 (52.5%)	197 (56.4%)	0.13562^b^
Male	68 (39.5%)	84 (47.5%)	152 (43.6%)	
Race				
White	151 (87.8%)	156 (88.6%)	307 (88.2%)	0.8067^b^
No White	21 (12.2%)	20 (11.4%)	41 (11.8%)	
Income				
< 1.450,00	133 (77.3%)	105 (62.1%)	238 (69.8%)	0.0022^b^
> 1.450,00	39 (22.7%)	64 (37.9%)	103 (30.2%)	
Marital Status				
Married	115 (66.9%)	98 (58.0%)	213 (62.5%)	0.0907^b^
Single	57 (31.1%)	71 (42.0%)	128 (37.5%)	
BMI				
Mean	25.6 (4.0)	26.4 (5.0)	26.0 (4.6)	0.1821^a^
Median	25.3	25.5	25.4	
Education (yr)				
< 8	145 (84.3%)	131 (74.0%)	276 (79.1%)	0.0181^b^
> 8	27 (15.7%)	46 (26.0%)	73 (20.9%)	
Smoking				
No	100 (58.1%)	73 (41.2%)	173 (49.6%)	0.0016^b^
Yes	72 (41.9%)	104 (58.8%)	176 (50.4%)	
Sun exposure				
No	19 (11.0%)	48.0 (27.3%)	67 (19.3%)	0.0001^b^
Yes	153 (89.0%)	128 (72.7%)	281 (80.7%)	
Systemic diseases				
No	42 (24.6%)	42 (23.7%)	84 (24.1%)	0.8560^b^
Yes	129 (75.4%)	135 (76.3%)	264 (75.9%)	
Employment status				
Employed	15 (8.7%)	52 (29.4%)	67 (19.2%)	< 0.0001^b^
Unemployed	157 (91.3%)	125 (70.7%)	282 (80.8%)	
Phisical activity				
No	105 (61.0%)	108 (61.0%)	213 (61.0%)	0.9955^b^
Yes	67 (39.0%)	69 (39.0%)	136 (39.0%)	
Ethylism				
No	155 (90.1%)	141 (80.1%)	296 (85.1%)	0.0222^b^
Yes	17 (9.9%)	35 (19.9%)	52 (14.9%)	
Vegetables				
Twice a week	19 (11%)	48 (27.3%)	67 (19.3%)	0.0001^b^
Less than twice	153 (89.0%)	128 (72.7%)	281 (80.7%)	

*BMI* Body mass index, *SD* Standart deviation, *BEVA* Best eye visual acuity

^a^Mann Whitney, ^b^Qui Quadrado

Comparisons of the clinical and demographic characteristics between the AMD and control groups suggested that patients with AMD were older, had higher income, were more likely to be employed, were more highly educated, and were more likely to be smokers than patients without AMD (Table 1).

Table 2 summarizes the mean differences in the NEI-VFQ-25 scores between the control and AMD groups. The mean composite score of the NEI-VFQ-25 questionnaire was 53.7 for the AMD group and 64.1 for the control group (p < 0.0001). All questionnaire subscales had lower scores in the AMD group compared to the control group, and most of these differences, except for dependency, driving, and color vision, reached statistical significance (p < 0.05).

**Table 2:** Mean NEI-VFG-25 scores of AMD and control groups

Domain (Mean ± SD (N))	AMD	Control	p-value
General_health	41.5 ± 21.1 (N = 177)	50.0 ± 14.0 (N = 172)	< .0001^a^
General_vision	39.9 ± 21.9 (N = 176)	53.4 ± 14.4 (N = 172)	< .0001^a^
Ocular_pain	65.4 ± 27.8 (N = 177)	78.4 ± 23.6 (N = 172)	< .0001^a^
Near_activities	44.8 ± 26.9 (N = 177)	55.3 ± 14.9 (N = 172)	< .0001^a^
Distance_activities	55.6 ± 26.3 (N = 177)	63.0 ± 18.3 (N = 172)	0.0020^a^
Social_functioning	69.6 ± 26.6 (N = 177)	79.1 ± 19.4 (N = 172)	0.0028^a^
Mental_health	33.8 ± 32.8 (N = 177)	41.0 ± 24.1 (N = 172)	0.0022^a^
Role_dificulties	35.5 ± 30.6 (N = 177)	50.7 ± 16.2 (N = 172)	< .0001^a^
Dependency	51.6 ± 36.3 (N = 177)	59.6 ± 27.4 (N = 172)	0.0728^a^
Driving	64.5 ± 29.3 (N = 50)	60.8 ± 20.5 (N = 36)	0.4649^a^
Color_vision	76.1 ± 28.0 (N = 176)	82.8 ± 20.3 (N = 172)	0.1126^a^
Peripheral_vision	64.1 ± 28.2 (N = 175)	78.4 ± 23.9 (N = 171)	< .0001^a^
Composite_score	53.7 ± 22.3 (N = 177)	64.1 ± 13.9 (N = 172)	< .0001^a^

^a^ Mann–Whitney

We separately evaluated the socioemotional and visual function components with their respective subscales and items to assess the psychometric constructs using the Rasch-calibrated NEI-VFQ-25 scores. The median score of the socioemotional component was 62.06 for the AMD group and 65.51 for the control group, with a difference of 2.90 (p = 0.0037). The median score of the visual function component was 56.41 for the AMD group and 61.53 for the control group, a difference of 4.00 (p = 0.0001).

Table 3 shows the differences in the mean VRQoL scores among the subgroups. It can be seen that subgroup 1 had the highest scores, followed by subgroups 2 and 3. In Subgroup 1, the three lowest score were role difficulties (43.2), general vision (45.3), and mental health (46.0). Subgroup 3 had lower scores for the subscales of mental health (18.8), role difficulties (25.0), and near activities (30.9).

**Table 3:** Mean NEI-VFQ 25 scores between subgroups of AMD

Domain (Mean ± SD (N))	Subgroup 1	Subgroup 2	Subgroup 3	p-value
General_health	38.2 ± 21.4 (N = 68)	45.4 ± 19.2 (N = 65)	40.9 ± 22.9 (N = 44)	0.2457^a^
General_vision	45.3 ± 23.3 (N = 68)	38.2 ± 21.7 (N = 65)	34.0 ± 17.7 (N = 43)	0.0186^a^
Ocular_pain	65.1 ± 26.9 (N = 68)	62.5 ± 28.4 (N = 65)	70.2 ± 28.2 (N = 44)	0.2457^a^
Near_activities	53.3 ± 26.0 (N = 68)	45.4 ± 26.6 (N = 65)	30.9 ± 23.2 (N = 44)	< .0001^a^
Distance_activities	63.7 ± 23.8 (N = 68)	56.6 ± 26.2 (N = 65)	41.5 ± 25.1 (N = 44)	< .0001^a^
Social_functioning	78.7 ± 23.8 (N = 68)	72.9 ± 21.3 (N = 65)	50.9 ± 29.1 (N = 44)	< .0001^a^
Mental_health	46.0 ± 33.3 (N = 68)	31.2 ± 32.0 (N = 65)	18.8 ± 25.9 (N = 44)	< .0001^a^
Role_dificulties	43.2 ± 30.3 (N = 68)	34.6 ± 31.6 (N = 65)	25.0 ± 26.6 (N = 44)	0.0055^a^
Dependency	64.3 ± 33.4 (N = 68)	50.9 ± 34.6 (N = 65)	32.8 ± 35.3 (N = 44)	< .0001^a^
Driving	66.7 ± 27.7 (N = 27)	57.8 ± 31.9 (N = 16)	71.4 ± 30.4 (N = 7)	0.5289^a^
Color_vision	84.3 ± 23.0 (N = 67)	76.9 ± 27.0 (N = 65)	62.5 ± 31.7 (N = 44)	0.0007^a^
Peripheral_vision	72.4 ± 25.8 (N = 67)	65.6 ± 27.6 (N = 64)	49.4 ± 27.2 (N = 44)	< .0002^a^
Composite_score	61.6.4 ± 20.2 (N = 68)	53.4 ± 21.19 (N = 65)	41.8 ± 21.1 (N = 44)	< .0001^a^

^a^ kruskal–wallis

Separate analyses of the effects of sociodemographic characteristics on the QoL of patients with AMD were performed using ANOVA to transform the domains of the NEI-VFQ-25 questionnaire into ranks. The sociodemographic and ocular characteristics that had the largest effects on the NEI-VFQ-25 score were family income, educational level, descent, diet (vegetables/fruits), physical activity, and VA. Marital status, sun exposure, alcoholism, and the presence of associated systemic diseases did not affect the responses to the NEI-VFQ-25 questionnaire.

AMD patients with higher family incomes scored significantly better on various subscales of the questionnaire, as well as the composite score (59.10 versus 50.30, p = 0.022), compared to those with lower incomes. AMD patients with higher educational levels also had higher composite scores than those with lower levels of education (60.36 versus 51.33, p = 0.013).

Participants of Brazilian descent had a composite score of 53.19, while participants of European descent had a composite score of 50.25 (p = 0.027). The habit of eating fruits and vegetables at least twice weekly was associated with a higher composite score of 55.15 compared to 45.17 for patients who consumed them less frequently (p = 0.039). Engaging in physical activity at least twice weekly was associated with a higher composite score of 59.55 compared to 49.92 for patients who exercised less frequently (p = 0.019).

The logarithm of the minimum angle of resolution (logMAR) VA affected six NEI-VFQ-25 subscales significantly and positively: dependence (− 0.32, p < 0.000), near activities (− 0.29, p = 0.002), role difficulties (− 0.28, p = 0.001), mental health (− 0.27, p = 0.011), color vision (− 0.24, p = 0.017), and composite score (− 0.30, p = 0.0027).

The longitudinal component of the study assessed a different group of 48 patients with exudative disease after three loading doses of anti-VEGF treatment. These patients had a mean age of 72.9 years, 56% were male, 62% were smokers, 56% engaged in physical activity less than twice weekly, and 79% had a history of sun exposure.

The mean logMAR VA of the treated eyes was 1.07 ± 0.44 (Snellen equivalent of 20/200 in both eyes) at baseline, and the mean logMAR VA after 4 months of follow-up was 0.92 ± 0.51 (Snellen equivalent of 20/160 in both eyes). The mean change in logMAR VA in treated eyes was a decrease of 0.16 (p = 0.01). The mean macular thickness measured on OCT at baseline was 335.28 μm, which decreased to 298.53 μm after four months of follow-up. The mean change in the OCT macular thickness was a decrease of 36.74 μm (p = 0.012).

The mean NEI-VFQ-25 subscale scores improved from baseline to the 4-month follow-up significantly in almost all subscales, except for the subscales that were not sensitive to AMD (i.e., general health, ocular pain, and color vision). The mean composite score variance increased by 11.17 points (p < 0.0001) (Table 4).

**Table 4:** Variance between baseline and month 4 follow up

Domain (Mean ± SD (N))	Baseline	Month 4 follow up	Mean*	P-value**
General_ health	43.75 ± 21.57 (N = 48)	44.79 ± 21.24 (N = 48)	1.04	0.8848
General_ vision	40.83 ± 21.2 (N = 48)	55.00 ± 22.03 (N = 48)	14.17	0.0004
Ocular_ pain	65.63 ± 26.86 (N = 48)	66.67 ± 27.81 (N = 48)	1.04	0.9286
Near_ activities	40.28 ± 22.17 (N = 48)	56.68 ± 24.38 (N = 48)	16.41	< .0001
Distance_ activities	52.69 ± 25.46 (N = 48)	61.98 ± 26.36 (N = 48)	9.29	0.0242
Social _functioning	63.54 ± 26.02 (N = 48)	77.60 ± 23.20 (N = 48)	14.06	0.0011
Mental_ health	27.73 ± 29.82 (N = 48)	50.95 ± 31.66 (N = 48)	23.22	< .0001
Role_ dificulties	38.28 ± 26.97 (N = 48)	48.18 ± 31.89 (N = 48)	9.9	0.0489
Dependency	48.61 ± 33.21 (N = 48)	68.75 ± 35.21 (N = 48)	20.14	< .0001
Driving	70.83 ± 14.43 (N = 03)	90.63 ± 6.25 (N = 04)	–***	–***
Color_ vision	73.44 ± 24.95 (N = 48)	83.33 ± 22.68 (N = 48)	9.9	0.0124
Peripheral_ vision	64.36 ± 26.45 (N = 47)	71.28 ± 23.88 (N = 47)	6.91	0.0960
Composite_ Score	50.92 ± 18.07 (N = 48)	62.42 ± 19.01 (N = 48)	11.5	< .0001

^*^ Diference Between Baseline and Month 4 follow up

^**^ Wilconson test for paired sample. Null hypothesis: median is zero

^***^ Frequency is less than enough to calculate

## Discussion

The current study reported data regarding VRQoL in a group of Brazilian patients with AMD and assessed the epidemiological and sociodemographic characteristics of the participants, which included patients suffering from all degrees of AMD severity. We evaluated how AMD affected VA and HRQoL using not only the traditional NEI-VFQ-25 analysis but also results calibrated with a separate Rasch analysis; the Rasch analysis separated the socioemotional component from the visual function component. We also evaluated the VRQoL in patients with macular neovascularization secondary to AMD at the time of enrollment and after three loading doses of anti-VEGF therapy.

Previous studies have investigated QoL in Brazilian patients with AMD using different methodologies. Marbac et al. conducted a cross-sectional study in which they assessed the HRQoL of patients who were legally blind due to advanced AMD and found that patients who were legally blind in both eyes had lower NEI-VFQ-25 scores than those who were legally blind in only one eye; nonetheless, both of these groups had lower scores than control subjects without ophthalmologic disease [17]. Pradella et al. investigated the epidemiological characteristics of 68 patients with AMD in southern Brazil in a cross-sectional study and reported that most were of European descent and that there was a higher incidence of advanced AMD compared to those of other studies [22]. Conversely, in the current study, most patients with AMD identified as being of Brazilian descent; this was likely due to the colonization of southern Brazil, resulting in this region having a higher proportion of people with European ancestry. Romani reported that AMD was the fifth most prevalent ophthalmologic disease in a southwestern Brazilian city [23].

Old age was the main risk factor for AMD in our sample, which is in concordance with the results of other studies conducted in other populations [24–27]. Cypel et al. studied a population of subjects aged 80 and older in São Paulo, Brazil, and found that AMD was the third most common cause of visual impairment [28]. Brazilian patients with AMD were more likely to have a higher income, a greater likelihood of being employed, and higher educational levels than the control patients. This patient profile is likely due to better socioeconomic conditions that lead to longer survival, and therefore, a greater chance of developing AMD [29]; therefore, older patients may be more socioeconomically privileged while also being more likely to develop AMD [25, 27, 28].

Based on the results in Table 1, there were more women in the AMD group than in the control group, but this difference did not reach significance. This result is consistent with the results of a study conducted in Brazil by Pradella et al. [22]. The Blue Mountains Eye Study and the Beaver Dam Eye Study studied non-Hispanic populations and described a higher frequency of AMD among women that was also not statistically significant [22, 24, 25]. In the current study, smoking was a risk factor for AMD, which corroborated previous studies [24–26] and highlighted the importance of counseling patients to quit smoking.

When analyzing the results of the NEI-VFQ-25 questionnaire, we found that participants with AMD had significantly lower composite scores, as well as almost all subscale scores, than patients without AMD. This result is similar to several other studies from different continents [13, 30–32]. Comparing the results of composite scores among different populations, the mean scores for the controls and cases were 79.2 and 65.7, respectively, in a Swedish population [32], and 91.1 and 75.1, respectively, in a Greek population [30]; the mean scores in the current study were 64.1 and 53.7, respectively. Although our results are comparable to those of other populations, the numbers of controls and cases in our study were lower than those of the other populations. This may reflect the difficulties in accessing health care that make this population more prone to visual loss and more susceptible to compromised VRQoL [13]. Although our patients had received treatment, their adherence to ophthalmologic appointments was not ideal. The lack of information about the disease course and the presence of scheduling barriers due to deficiencies in the Brazilian public health system may have biased our results. Despite these issues, the current findings reflect the reality of the Brazilian public health system in the Campinas region.

The subscales most affected by the presence of AMD were mental health and role difficulties, and these results were similar to those of the EQUADE Study, a French nationwide cross-sectional observational survey that assessed VRQoL with the NEI-VFQ-25 questionnaire [8]. These results reflected the natural disease course in which a progressive or rapid loss of central vision led to changes in patients’ ability to perform daily activities and resulted in anxiety, increased dependency, and psychological distress.

Several investigators have used Rasch analysis as a newer psychometric method to validate and re-engineer the results of the NEI-VFQ-25 questionnaire and its subscales [21, 33]. A previous study in a Brazilian population suggested that the NEI-VFQ-25 questionnaire in isolation may not be psychometrically optimal for assessing QoL related only to visual function [21], and because of that, we also used Rasch analysis to obtain results that were less biased by the socioemotional component. When we evaluated the results of the Rasch-calibrated NEI-VFQ-25 questionnaire in the current study, the results reaffirmed the differences in the VRQoL between the AMD and control groups in not only the visual function component, but also in the socioemotional component.

The results analyzed in each AMD subgroup allowed a better understanding of the impact of AMD on the QoL of patients. A comparison of patients with early AMD (subgroup 1) versus controls showed that even early AMD can be associated with reductions in VRQoL. This is similar to what was reported in a Latino population in the Los Angeles Latino Eye Study, as we observed a similar correlation in the composite scores [13].

Patients with bilateral late AMD (subgroup 3) reported significantly worse NEI-VFQ-25 scores in almost all subscales compared to patients with unilateral late AMD (subgroup 2). This finding agreed with a Turkish study [31], in which the mean general vision subscale scores for bilateral and unilateral exudative AMD were 41.0 and 45.6, respectively (p < 0.001). Soubrane et al. also conducted a multicenter cross-sectional observational study that included patients from Canada, France, Germany, Spain, and the United Kingdom and reported a mean NEI-VFQ-25 composite score of 46 for patients with late bilateral AMD [34]. This agreed with our findings in patients with late bilateral AMD (subgroup 3), who had low NEI-VFQ-25 scores in almost all subscales, with a mean composite score of 41.8.

Our study evaluated how some sociodemographic and ocular characteristics can independently affect the VRQoL of patients with AMD. We observed that a higher educational level was associated with higher patient QoL. This socioeconomic characteristic was the only one that, besides the significant improvement in the subscale scores of distance activities, social functioning, mental health, and peripheral vision, also improved the driving subscale score. The driving subscale was only significant a few times, educational level and smoking habits, in the current analyses, because the sample comprised mostly participants who had never driven, and, therefore, the sample was too small to draw a conclusion.

We observed that a diet rich in fruits and vegetables was associated with significantly higher scores in several subscales. A previous study showed that adequate intake of fruits, vegetables, and fish reduced the risk of AMD [35], and yet another study reported that a healthy diet increased QoL [36]. These findings suggest that a healthy diet in conjunction with adequate treatment can alleviate the negative effects of AMD on HRQoL.

Regular physical activity improved the questionnaire scores in both the AMD-related subscales, such as near activity and dependence, and in the ocular pain subscale; this result agreed with other studies demonstrating that physical activity improves chronic pain and HRQoL [37, 38].

We used longitudinal analysis to better assess QoL, which is subjective, in addition to neovascular disease, improvement after treatment, and burdens. We assessed this objectively by evaluating the results of the NEI-VFQ-25 questionnaire before anti-VEGF treatment and after three loading doses. The analysis showed that the treatment improved not only the regular indicators of AMD, such as VA and macular thickness, but also the NEI-VFQ-25 score. Treated patients had significant improvements in the NEI-VFQ-25 scores in almost all subscales as well as the composite score. This result agreed with the ANCHOR study, a randomized clinical trial that used fluorescein angiography to compare ranibizumab (Lucentis, Genentech Inc., South San Francisco, CA, USA) with verteporfin (Visudyne, Bausch & Lomb, Rochester, NY, USA) photodynamic therapy in patients with predominantly classic choroidal neovascularization [39]. It was also in concordance with the MARINA study, another randomized clinical trial that compared monthly intraocular ranibizumab with sham injections for minimally classic/occult neovascular AMD; this study reported better VA, reduced macular thickness, and higher NEI-VFQ-25 scores after AMD treatment [40].

Moreover, the subscales for near activities, distance activities, and dependency, which are important assessments for patients with AMD [41, 42], improved significantly, and this is similar to the results reported by other studies [39, 40]. The mental health subscale score, while not directly related to AMD, also improved after treatment in the current study, as well as in other studies that assessed the effects of AMD treatment [39, 43]. This result was expected because it is known that patients with AMD have a higher prevalence of depressive disorders than healthy individuals [5]. After the three loading doses of anti-VEGF, the subscale scores for ocular pain, general health, and peripheral vision did not change significantly, which was similar to the results of other studies [40, 42]. This was also not surprising, as AMD is a painless central disease that does not directly impact general health.

The current study had several limitations. First, the study was limited to a specific region of Brazil and may not be generalizable to all Brazilian health systems and the current condition of AMD patients in other regions of Brazil. Second, the stratification of the patients into three different subgroups was done using an arbitrary classification system. However, it was useful in providing an assessment of the impact of AMD on VRQoL when comparing bilateral with unilateral disease. In addition, we included the CARMS classification as used in previous studies. Third, we did not apply other patient-reported outcome measures for comparison with the NEI-VFQ-25 findings, even though the NEI-VFQ-25 remains the most commonly used tool to evaluate VRQoL in ophthalmology studies. Another limitation was the use of a self-reported questionnaire to assess the socioeconomic and epidemiologic variables. However, previous studies have shown that self-reporting is accurate for variables such as alcohol intake [44] and systemic diseases [45]. Other studies have also reported the accuracy of self-reporting when evaluating different comorbidities in various ethnic groups [45, 46].

## Conclusion

This study describes the real-life burden caused by AMD and the effect of treatment on QoL in a Brazilian community. Patients with AMD were older, had higher incomes, were more likely to be employed, had higher education levels, were more likely to be smokers compared to patients without AMD. AMD patients tended to have poorer HRQoL than controls, especially with regards to the mental health and general difficulty subscales of the NEI-VFQ-25. The Rasch-calibrated NEI-VFQ-25 results revealed lower scores for the AMD group in both the visual function and socioemotional components.

The severity and bilaterality of AMD substantially reduced QoL, as reflected by the NEI-VFQ-25 scores. Conversely, higher family income and educational level, Brazilian descent, good dietary habits, and regular physical activity were positively correlated with NEI-VFQ25 scores. Patients with exudative AMD who were treated with three loading doses of anti-VEGF therapy had significant improvements in VA, macular thickness, and the composite and most subscale scores of the NEI-VFQ-25. These results will be important in influencing public health policy to improve health care access for AMD patients in Brazil.

## Data Availability

The dataset can be made available from corresponding author on request.

## Abbreviations

AMD: Age-related macular degeneration
OCT: Optical coherence tomography
HRQoL: Health-related quality of life
VRQoL: Vision-related quality of life
NEI-VFQ-25: National Eye Institute-Visual Function Questionnaire-25
VEGF: Vascular endothelial growth factor
VA: Visual acuity
logMAR: Logarithm of the minimum angle of resolution
QoL: Quality of life
CARMS: Clinical Age-Related Maculopathy Staging
